# Node Self-Deployment Algorithm Based on Pigeon Swarm Optimization for Underwater Wireless Sensor Networks

**DOI:** 10.3390/s17040674

**Published:** 2017-03-24

**Authors:** Shanen Yu, Yiming Xu, Peng Jiang, Feng Wu, Huan Xu

**Affiliations:** College of Automation, Hangzhou Dianzi University, Hangzhou 310018, China; shanen_yu@hdu.edu.cn (S.Y.); xymhdu@163.com (Y.X.); fengwu@hdu.edu.cn (F.W.); xuhuan@hdu.edu.cn (H.X.)

**Keywords:** node self-deployment, network layer, network cluster, pigeon swarm optimization

## Abstract

At present, free-to-move node self-deployment algorithms aim at event coverage and cannot improve network coverage under the premise of considering network connectivity, network reliability and network deployment energy consumption. Thus, this study proposes pigeon-based self-deployment algorithm (PSA) for underwater wireless sensor networks to overcome the limitations of these existing algorithms. In PSA, the sink node first finds its one-hop nodes and maximizes the network coverage in its one-hop region. The one-hop nodes subsequently divide the network into layers and cluster in each layer. Each cluster head node constructs a connected path to the sink node to guarantee network connectivity. Finally, the cluster head node regards the ratio of the movement distance of the node to the change in the coverage redundancy ratio as the target function and employs pigeon swarm optimization to determine the positions of the nodes. Simulation results show that PSA improves both network connectivity and network reliability, decreases network deployment energy consumption, and increases network coverage.

## 1. Introduction

Underwater wireless sensor networks (UWSNs) are network-monitoring systems in an underwater environment. The sensor nodes in these networks have perceptual, acoustic communication, and computational capabilities, and they can transmit the sensed information to the sink node for processing and analysis by one-hop or multi-hop methods [1,2]. UWSNs have been applied to water environment monitoring, underwater resource exploration, and marine military defense [3,4]. Research on UWSNs [5] mainly involves network architecture design [6], node deployment, node localization [7,8], time synchronization [9], network protocol design [10,11], etc. As the first step in UWSNs application, node deployment significantly affects network service in different aspects, including network coverage, network connectivity, and network energy consumption. The node-deployment problem is defined that move sensor nodes to the corresponding positions in an artificial or a self-organized manner to form a network topology that has special characteristics and increases the network service [4,12]. Meanwhile, the goals of node deployment are to increase network coverage and network connectivity, decrease network energy consumption, and prolong network lifetime. Generally, the problem of node deployment can be divided into static deployment, move-restricted node self-deployment, and free-to-move node self-deployment according to the mobility of the node [2,13,14,15,16,17]. In static deployment, the nodes cannot move and are deployed in the target area by the artificial method [14]. Thus, static deployment is not appropriate for the large-scale deployment. In move-restricted node self-deployment, the nodes can move only vertically and adjust their depth by themselves according to the local information near the node [18]. In free-to-move node self-deployment, the nodes have the capability to move freely in all directions [19]. With the improvement of the design level of the sensor node, the cost of the mobile nodes is reduced and more and more scholars concentrate on the research of free-to-move node self-deployment. Moreover, free-to-move node self-deployment is closer to the reality. Therefore, free-to-move node self-deployment has wide application scenarios and has important research significance.

The existing free-to-move node self-deployment algorithms [20,21,22,23,24,25] usually regard the event coverage as the research object, and aim at the network coverage, but these algorithms do not regard the network connectivity and network reliability as the goal simultaneously. Furthermore, in these algorithms, each node should move several rounds to determine the final deployed position, which increases the network deployment energy consumption. Consequently, there is still much space to improve the performance of free-to-move node self-deployment algorithms.

Thus, the current paper proposes the pigeon-based self-deployment algorithm (PSA) to solve these problems. The sink node first finds its one-hop nodes and maximizes the coverage in its one-hop region. This process allows for the maintenance of the number of the nodes deployed in the region near the sink node and increases network reliability and network coverage as much as possible. The one-hop nodes then divide the network into layers and cluster in every layer. This mechanism forms a distribution in which the scale of the cluster is small and the number of cluster head nodes is great in the region close to the sink node. The cluster head nodes do not move again, thus, the number of nodes in the region close to the sink node increases. Each cluster head node subsequently constructs a connected path to the sink node to guarantee network connectivity. Finally, the cluster head node regards the ratio of the movement distance of a cluster-in node to the change in the coverage redundancy ratio (CRR) of the node as the target function on the premise that the cluster-in node should be connected to the cluster head node by one-hop or multi-hop path. Pigeon swarm optimization is then employed to solve the moving position of the node. This method can determine the optimal deployment location in the continuous solution space, and minimize the complexity of the search for the optimal deployment location. Moreover, the moving position of each node can be determined at once, which can reduce the deployment energy consumption. PSA is compared with the three-dimensional virtual forces deployment algorithm (TVFDA) [25] in terms of network coverage, network connectivity, network reliability and energy consumption. The simulation results show that PSA increases both network connectivity and network reliability, decreases network energy consumption, and increases network coverage.

The rest of this paper is organized as follows: Section 2 introduces the related work about free-to-move node self-deployment. Section 3 describes the system model, preliminaries, and definitions considered in this study. Section 4 presents details of PSA. Section 5 analyses the complexity of PSA. Section 6 discusses the performance study and provides a detailed analysis of its result. Finally, Section 7 concludes the paper and presents future research directions.

## 2. Related Work

Some scholars have recently developed free-to-move node self-deployment algorithms for UWSNs. Wang et al. [20,21] proposed three kinds of movement-assisted sensor deployment algorithms, namely, vector-based algorithm, Voronoi-based algorithm, and Minimax algorithm. These three algorithms are all based on the Voronoi diagram. In the vector-based algorithm, a node moves to the next position according to the repulsive force exerted on the node by its Voronoi neighbor nodes. In the Voronoi-based algorithm, a node moves to the farthest vertex of its Voronoi diagram every round. In the Minimax algorithm, a node moves closer to the farthest vertex of its Voronoi diagram to avoid the situation in which a vertex that was originally close becomes the new farthest vertex. In these algorithms, each node can move autonomously to the corresponding position to cover the coverage hole according to its own local information. Thus, these algorithms can increase network coverage, and meet the distributed characteristic of the sensor network. However, the Voronoi diagram of each node should be formed in each of the iterations, and this process requires each node to know the global information of the network and consequently results in extra communication energy consumption. Moreover, the process based on the 3D Voronoi diagram of the node is more complex compared with that based on 2D Voronoi diagram. Thus, these algorithms are suitable for 2D node self-deployment but unsuitable for node self-deployment in UWSNs. Koutsougeras et al. [22] proposed a sensor deployment algorithm based on self-organizing maps for the event. In each of the iterations, an event is selected by a random sequence, and the node that is closest to the event should update its position according to the rule of movement. The node moves a certain distance to the event, and the distance of the movement is proportional to the distance between the node and the event. The algorithm is iterated until no more significant movement is possible. This algorithm can deploy the node according to the density of the event in the region and accomplish an uneven deployment in the target area. However, this algorithm adjusts the node position by the centralized way, which is difficult to achieve in practice. Xia et al. [23] proposed a fish swarm-inspired underwater sensor deployment algorithm (FSSDA). Drawing on the foraging behavior of the fish swarm, this algorithm regards the node as the fish and the event as the food, moves the node to cover the event, and finally completes the deployment for the event according to the crowding parameter of each event. The algorithm has low computational complexity and rapid convergence rate. Du et al. [24] proposed a particle swarm-inspired underwater sensor self-deployment algorithm. The process of the node deployment in the algorithm is similar to that in FSSDA, but the algorithm also considers the situation in which the position of the event is dynamically changing, like the situation in practice. However, both algorithms are suitable for the distributed coverage of the event but unsuitable for the coverage of the region. In addition, these algorithms also ignore network connectivity. Li et al. [25] proposed a three-dimensional virtual forces deployment algorithm (TVFDA). This algorithm applies the concepts of gravitation and repulsion in physics to the wireless sensor network and considers that these forces exist between nodes in the network. Therefore, in the algorithm, each node moves to the next position according to the join forces, which are exerted on the node by its neighbor nodes, and each node stops moving until its join forces is zero or the maximum iteration number is reached. This algorithm allows for an even distribution of the nodes in the target area compared with situation before the algorithm is run; thus, this algorithm enhances network coverage and meets the characteristics of underwater wireless sensor networks. However, in this virtual force algorithm, each node needs to know the related information of its neighbors in each iteration, that is, each node should communicate with its neighbor nodes in each iteration; thus, this algorithm results in increased network energy consumption. Moreover, each node should move a real distance several times according to the gravitational and repulsive forces exerted on it. Consequently, this algorithm may segment the network, degrade network connectivity, and increase network energy consumption for the node deployment. Furthermore, being incapable of controlling the number of the nodes in the region near the sink node, TVFDA may degrade both network reliability and energy efficiency.

## 3. Preliminaries, Models, and Definitions

### 3.1. Preliminaries

*N* nodes are thrown in the target area (rectangular or cubic) uniformly and randomly. Each node then moves to the determined position with the aid of an autonomous underwater vehicle (AUVs), and each node is fixed at the position by an anchor after its deployment position is calculated by the deployment algorithm. In addition, sensor nodes communicate with one another through acoustic channels and maintain connectivity with the sink node via one-hop or multi-hop paths. The following assumptions are made:

(1) A node adopts the Boolean sensing model [26], and the sensing radius of the node is fixed.
(2) All nodes have the same states, including initial energy, sensing radius, and communication radius, before the node deployment. Moreover, the communication radius of the node can be adjusted according to the demand of the algorithm but should not exceed the maximum communication radius, $R_{c}^{\max}$ which is determined by the physical device during the node deployment.
(3) With the similar assumptions in [24] as inspiration, the sink node is fixed at the center of the water face. By contrast, the other nodes can move freely in all directions, and their real-time locations can be determined using a localization algorithm or global positioning satellite devices during the node deployment.

### 3.2. Related Models

#### 3.2.1. Coverage Redundancy Ratio

The coverage redundancy ratio (CRR) of the node (i.e., *s_i_*) is defined as the ratio of the sensing area of the node with its neighbor node (i.e., the node within *s_i_*’s maximum communication radius) to *s_i_*’s sensing area [27]. CRR is formulated as follows [27]:

(1) $$\gamma (s_{i})=1-\prod_{j=1}^{n}\left(1-\frac{2\left[\frac{2}{3}\pi \left(R_{s}^{3}-\frac{d^{3}(s_{i},s_{j})}{8}\right)-\frac{\pi d(s_{i},s_{j})}{2}\left(R_{s}^{2}-\frac{d^{2}(s_{i},s_{j})}{4}\right)\right]}{\frac{4}{3}\pi R_{s}^{3}}\right)$$

where *n* is the number of the neighbor nodes of node *s_i_* and *d*(*s_i_,s_j_*) is the distance between node *s_i_* and its neighbor node *s_j_*.

#### 3.2.2. Underwater Energy Consumption

During the node deployment, each node should determine the local information near its position by communicating with its neighbor nodes, and each node should move to the determined position when it knows the position. Therefore, the energy consumption in this study is divided into communication energy consumption and deployment energy consumption.

(1) Communication Energy Consumption

The nodes of an underwater sensor network communicate with one another by acoustic signals [28]. Thus, the communication energy consumption of the acoustic medium [29] is adopted in this study and given by

(2) $$E_{c}\left(d\left(s_{se},s_{rec}\right)\right)=P_{r}\times T_{p}\times A\left(d\left(s_{se},s_{rec}\right)\right)$$

This expression describes the energy consumption when the data packet is transmitted from the sending node to the receiving node. In Equation (2), *P_r_* is the minimum number of power packets that can be received, and *T_p_* is the data transmission time, whose formulation is as follows:

(3) $$T_{p}=\frac{L_{b}}{V_{t}}$$

where *L_b_* is the length of the sent data packet and *V_t_* is the transmission speed of the data packet in water. The energy attenuation during the transmission of the data packet at a given distance is described as follows:

(4) $$A\left(d\left(s_{se},s_{rec}\right)\right)=d{\left(s_{se},s_{rec}\right)}^{\lambda }\cdot \alpha^{d\left(s_{se},s_{rec}\right)}$$

where *λ* is the energy diffusion factor, whose value is generally equal to 1.5, and *α* is a parameter determined by the following expression:

(5) $$\alpha =10^{a\left(F_{r}\right)/10}$$

In Equation (5), *a*(*F_r_*) is the absorption coefficient (in dB/m) formulated as follows:

(6) $$a\left(F_{r}\right)=0.11\frac{10^{-3}F_{r}^{2}}{1+F_{r}^{2}}+44\frac{10^{-3}F_{r}^{2}}{4100+F_{r}^{2}}+2.75\times 10^{-7}F_{r}^{2}+3\times 10^{-6}$$

where *F_r_* is the carrier frequency in kHz.

(2) Deployment Energy Consumption

In the process of the node self-deployment for underwater wireless sensor networks, each node moves to the determined position that the node self-deployment algorithm calculates. The energy consumption because of node moving in the process is called the deployment energy consumption. Because the methods of nodes moving are various, it is difficult to express with a specific model. Therefore, in the problem of node deployment, the total distance of all nodes in the deployment process is used to measure the deployment energy consumption of a node deployment algorithm.

### 3.3. Definitions

#### 3.3.1. Network Coverage

**Figure 1:**
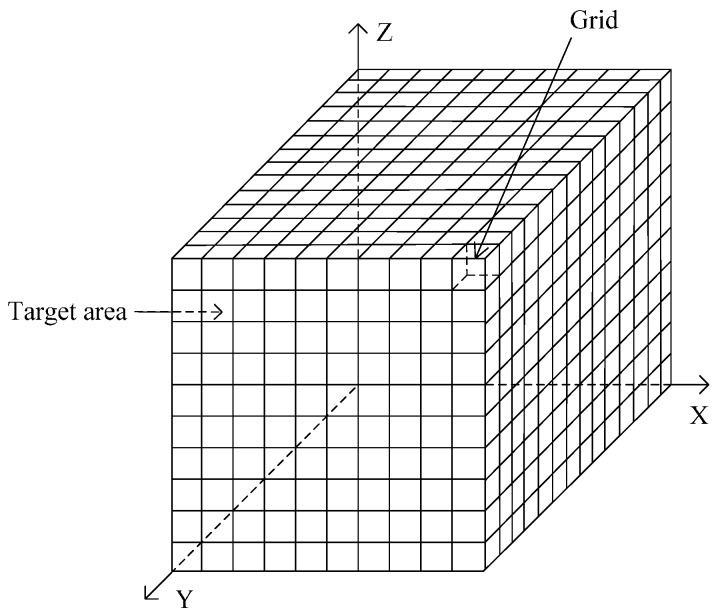
Underwater 3D space model.

An underwater 3D space (target coverage area) is usually represented by a cuboid or cube. This space is divided into a number of small cubic grids whose sides have the length *w* [18]. The center of each cubic grid called the grid point, which is regarded as the representative of the grid. Each grid point has its own coordinates. A typical underwater 3D space is illustrated in Figure 1.

Network coverage, which reflects the degree an underwater sensor network covers a monitoring or target area, is a primary criterion parameter in the evaluation of a node-deployment algorithm. This parameter is defined as the ratio of the number of grid points covered by UWSNs to the total number of grid points in the target area. Network coverage is denoted by *r_cov_*, whose formula is as follows:

(7) $$r_{\mathrm{cov}}=\frac{N_{\mathrm{cov}}}{N_{total}}$$

where *N_cov_* is the number of grid points covered by the active nodes and *N_total_* is the total number of grid points in the target area.

#### 3.3.2. Network Connectivity

Network connectivity is another important criterion parameter for evaluating the quality of service in sensor networks. This parameter is a prerequisite for the effective application of sensor networks. It is defined as the ratio of the number of nodes connected to the sink node by a one-hop or multi-hop path to the total number of nodes in the network. The number of nodes connected to the sink node can be calculated by breadth-first search, in which the sink node is the root node.

## 4. Problem and Algorithm Description

### 4.1. Problem Description

The existing free-to-move node self-deployment algorithms usually aim at the event coverage and deploy the node according to the network coverage. However, these algorithms do not consider the problems related to network connectivity, network reliability, and network energy consumption. Furthermore, these algorithms determine the final position of each node after the node moves several rounds according to the result of every iteration of the algorithm. This process induces each node to move to the final position not in a straight line, thereby increasing the moving distance of the node and the energy consumption. In these algorithms, the network coverage is increased by forcing each node to move an appropriate distance in each of the iterations so that the node can stay away from each other as far as possible. This process decreases the area of the overlapping region in the target area; however, the probability of deploying the node near the sink node is also decreased. As a result, holes appear in the locations near the sink node more easily, and the network service may be degraded regardless of the network routing protocol. In addition, positioning the nodes far from other nodes, may lead to a segmented network, which detrimentally also affects network connectivity. Thus, in this study, the problem is defined as follows: given *N* sensor nodes that are thrown randomly and uniformly in the deployment space, design an algorithm to maximize network coverage, maintain network connectivity while minimizing energy consumption.

This study proposes the pigeon-based self-deployment algorithm (PSA) for UWSNs to solve the drawbacks of the existing free-to-move node self-deployment algorithms. In PSA, the sink node first finds its one-hop nodes and maximizes network coverage in its one-hop region. The one-hop nodes then divide the network into layers and cluster in each layer. Each cluster head node constructs a connected path to the sink node to guarantee network connectivity. Finally, each cluster head node regards the ratio of the movement distance of the node to the change in CRR of the node as the target function and uses pigeon swarm optimization to solve its position. As a result, the network coverage is expanded, the deployment energy consumption is reduced, and the number of node in the region near the sink node is increased.

### 4.2. Algorithm Description

#### 4.2.1. Pigeon Swarm Optimization Algorithm

The pigeon swarm optimization algorithm (PSOA) is proposed by Duan and Quiao [30,31] in 2014. The algorithm is derived from the behavior of homing pigeons. PSOA consists of two models, namely, the map and compass operator and the landmark operator. PSOA has better optimization performance and faster convergence speed, comparing with other artificial intelligence algorithms [32,33].

(1) Map and Compass Operator

When all the pigeons are not familiar with the destination or landmark during the initialization of the algorithm, they determine the flying position and direction according to the magnetic field and the position of the sun. In this model, each pigeon updates its position according to the recent global optimal solution in the current iteration. On the assumption that the position and speed of the *j*-th pigeon are *X_j_* and *V_j_*, respectively, *X_j_* and *V_j_* are updated according to Equations (8) and (9) in the *t*-th iteration.

(8) $$V_{j}\left(t\right)=V_{j}\left(t-1\right)\cdot e^{-Gt}+rand\cdot \left(X_{g}-X_{j}\left(t-1\right)\right)$$

(9) $$X_{j}\left(t\right)=X_{j}\left(t-1\right)+V_{j}\left(t\right)$$

where *G* is the map and compass factor, *rand* is a random number, and *X_g_* is the global optimal position in the *t*-th iteration.

(2) Landmark Operator

After PSOA has run for some time, some pigeons may have found the destination or familiar landmarks. Thus, these pigeons can move to the destination quickly, and the others move behind them. On the assumption that *X_c_*(*t*) is the center of the position of the pigeon whose fitness is the top *N_p_*/2, the position of each pigeon in the *t*-th iteration is

(10) $$N_{p}\left(t\right)=\frac{N_{p}\left(t-1\right)}{2}$$

(11) $$X_{c}\left(t\right)=\frac{\sum X_{j}\left(t\right)\cdot fitness\left(X_{j}\left(t\right)\right)}{\sum fitness\left(X_{j}\left(t\right)\right)}$$

(12) $$X_{j}\left(t\right)=X_{j}\left(t-1\right)+rand\cdot \left(X_{c}\left(t\right)-X_{j}\left(t-1\right)\right)$$

where *N_p_*(*t*) is the number of pigeons that meets the restriction of the condition in the *t*-th iteration and *fitness*(*X_j_*(*t*)) is the proportion of the fitness of the *j*-th pigeon to that of all the pigeons. In the minimum optimization problem, the fitness is formulated as follows:

(13) $$fitness\left(X_{j}\left(t\right)\right)=\frac{1}{f\left(X_{j}\left(t\right)\right)+\varepsilon }$$

where, *f* is the fitness function andis a small value.

#### 4.2.2. PSA Process Description

The process of PSA is divided into three stages: (1) deploying in the one-hop region of the sink node; (2) network layering and clustering; and (3) optimizing the position of the cluster-in nodes.

(1) Deploying in the one-hop region of the sink node

After the nodes are thrown in the target area uniformly and randomly, the sink node broadcasts “beginning deployment” information with the communication radius, $R_{c}^{\min}$. The node receiving the information (i.e., *s_i_*) confirms the sink node as its next hop node and relays its location information, *p_original_*(*s_i_*):

(14) $$p_{original}\left(s_{i}\right)=\left[x_{original}\left(s_{i}\right),\;y_{original}\left(s_{i}\right),\;z_{original}\left(s_{i}\right)\right]$$

These nodes are called the one-hop nodes of the sink node, or the one-hop node *SH*. |*SH*| is the number of elements in the set of one-hop nodes. The sink node then optimizes the network coverage in the one-hop region of the sink node. The ratio of the deployment energy consumption for moving the node to the final position, *p_deployed_*(*s_i_*), to the difference between CRR in *p_original_*(*s_i_*) and *p_deployed_*(*s_i_*) is regarded as the fitness function *fitness*_1_:

(15) $$\min\;fitness_{1}=\left\{ \begin{array}{ll} \frac{\sum_{s_{i}\in SH}d\left(p_{deployed}\left(s_{i}\right),p_{original}\left(s_{i}\right)\right)}{\sum_{s_{i}\in SH}\gamma \left(p_{original}\left(s_{i}\right)\right)-\gamma \left(p_{deployed}\left(s_{i}\right)\right)} & \mathrm{denominator}\;\mathrm{is}\;\mathrm{greater}\;\mathrm{than}\;0\\ \inf & \mathrm{else} \end{array}\right.$$

(16) $$\begin{array}{ll} s.t. & d\left(\sin k,\;p_{deployed}\left(s_{i}\right)\right)\leq R_{c}^{\min}\\ & z_{deployed}\left(s_{i}\right)<z\left(\sin k\right)-c_{border}\;\forall s_{i}\in SH \end{array}$$

where *c_border_* is a constant expressing the minimum distance between the deployment position of the node and the boundary of the target area.

**Figure 2:**
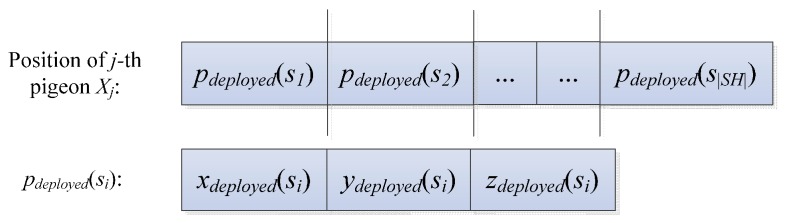
Solution coding diagram.

The number of the pigeons is set to *N_p_*, and the positions of the pigeons are initialized. The position of the *j*-th pigeon, *X_j_*, consists of the coordinates of |*SH*| random points, each of which represents the position of a node in the set *SH*. These random nodes are in the hemisphere that regards the sink node as the center of the sphere. These nodes also regard the radius of the sphere to be 1.25 times that of the minimum communication radius. Thus, *X_j_* is illustrated in Figure 2.

Subsequently, the solution of *fitness*_1_, *X_g_*, is calculated according to the PSOA described in Section 4.2.1, and the position of node *s_i_* is formulated as follows:

(17) $$p_{deployed}\left(s_{i}\right)=\left[\begin{array}{ccc} X_{g}\left[3i-2\right] & X_{g}\left[3i-1\right] & X_{g}\left[3i\right] \end{array}\right]$$

(2) Network Layering and Clustering

This stage is further divided into four steps.

Step 1. After the one-hop node *s_i_* moves to the calculated position, all the other one-hop nodes set their layer number, *level*(*s_i_*), to 1; the corresponding layer width, *width*(*level*(*s_i_*)), to the initial communication radius, $R_{c}^{\min}$; and the broadcast radius, *R_b_*(*s_i_*), to $R_{c}^{\min}$. Then, they broadcast their information, (ID, *level*(*s_i_*)).
Step 2. The node *s_j_* receiving the information compares the layer number with that of *s_i_*. If the layer number is greater than that of *s_i_*, then *level*(*s_j_*) is set to *level*(*s_i_*) + 1 and *R_b_*(*s_j_*), and *width*(*level*(*s_j_*)) are set to *R_b_*(*s_i_*).
Step 3. The node *s_j_* broadcasts the information *level*(*s_j_*), and the nodes having the same layer number as *s_j_* reply the distance between the sink node and themselves to *s_j_*. Then, *s_j_* compares the distance with its distance to the sink node, *d*(*s_j_*,*Sink*), to determine if *d*(*s_j_*,*Sink*) is the minimum value among the distances of the nodes relaying the distance to *s_j_*. Consequently, *s_j_* becomes a cluster head node and broadcasts the information *M_c_*. By contrast, *s_j_* enters the stage of waiting message. In time *T*, if *s_j_* receives *M_c_*, then *s_j_* becomes a common node and broadcasts *M_nc_*. If *s_j_* receives *M_nc_*, then *s_j_* ignores the distance a node sent and compares its distance to the sink node with that of the nodes, except the node sending *M_nc_*. *s_j_* also becomes the cluster head node when *s_j_* waits for time *T*. The common node then joins into the cluster, which is the closest to it, and the nodes in current layer update the broadcast radius *R_b_*(*s_j_*),
  (18) $$R_{b}\left(s_{i}\right)=\min\left(width\left(level\left(s_{i}\right)\right)+width\left(level\left(s_{i}\right)-1\right),R_{c}^{\max}\right)$$
Step 4. Each node *s_j_* in the current layer broadcasts the information (*level*(*s_j_*), cluster head node or not). For the node receiving the information, the node whose layer number is greater than *level*(*s_j_*) proceeds to Step 2 and clusters with the residual nodes. The node whose layer number is less than *level*(*s_j_*), relays the information (self is cluster head node or not) to the cluster head node and ignores the information the common nodes send. The cluster head node in the current layer selects the closest cluster head node in the last layer as the next-hop node. If it does not receive the information of the cluster head node in the last layer, then it selects the closest common node in the last layer as the next-hop node.

After a certain time, if the node has the initial layer number, then it broadcasts its maximum communication radius and enters the closest cluster. If the node has the initial layer number and only has neighbor nodes with the initial layer number in its maximum communication radius, then this node moves a distance in the direction of the sink node one time and subsequently finds its next-hop node. Distance (signed as *d_sink_* in following paper) here refers to the average distance between the node and its closest node in the direction of the sink node minus the maximum communication radius.

**Figure 3:**
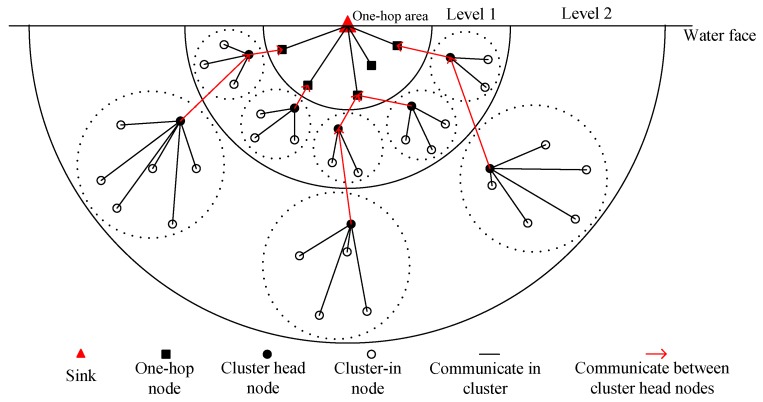
Network Distribution Diagram.

After Stage (2) is run, the network distribution is generally shown in Figure 3. The number of the cluster in the region near the sink node is greater than that in the region far from the sink node.

**Figure 4:**
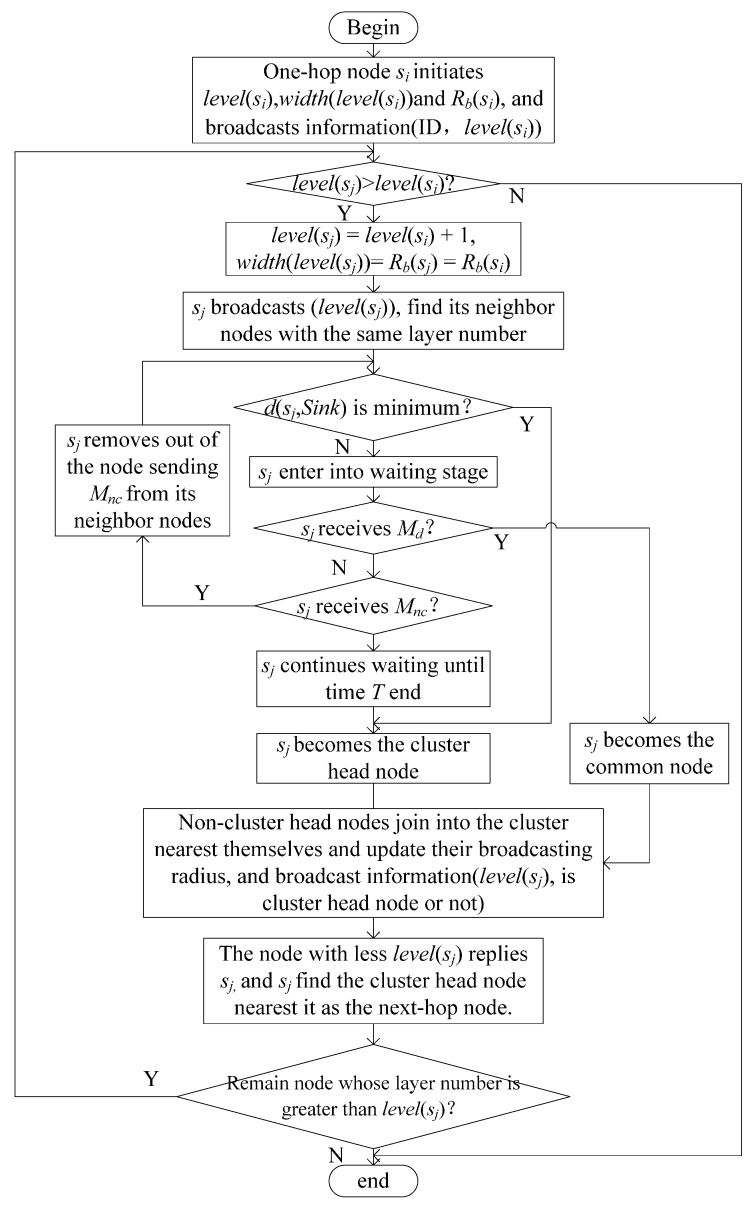
Flowchart of network layering and clustering.

The flowchart of network layering and clustering is presented in Figure 4.

(3) Optimizing the Position of the Cluster-in Nodes

After Stage (2), each cluster node (i.e., *s_k_*) knows its own cluster-in node set *S_c_*(*s_k_*) and their corresponding original positions *P_o_*(*s_k_*). When the cluster head node, *s_k_*, receives the command “calculating deployment location”, it calculates the deployment position of its cluster-in nodes.

First, *s_k_* updates the set of nodes whose deployment positions have been calculated in the network *S_ND_* and the set of nodes whose deployment positions have been calculated in its cluster *S_CD_* (the element in *S_CD_* is only the cluster head node when the cluster head node begins to optimize the positions of its cluster-in nodes). Subsequently, *s_k_* calculates the deployment positions of its cluster-in nodes by using PSOA, and the deployment position of each cluster-in node is determined using PSOA once. The detailed procedure follows.

(1) Selecting Next Node to be Calculated

The cluster head node *s_k_* calculates the CRR values of its cluster-in nodes whose deployment positions in the network have not yet been calculated and then selects the node (i.e., *s_i_*) with the minimum CRR as the next node to be calculated.

(2) Constructing the Fitness Function

The cluster head node *s_k_* firstly formulizes the deployment energy consumption that nodes move from the initial position to the deployment position and subsequently formulizes the increase in the network coverage. Finally, *s_k_* regards the ratio of the deployment energy consumption to the increase in the network coverage as the fitness function *fitness*_2_ on the premise that *s_i_* can connect with at least one node in *S_CD_* after *s_i_* moves to the deployment position. The fitness functions *fitness*_2_ is formulated as follows:

(19) $$\min\;fitness_{2}=\left\{ \begin{array}{ll} \frac{d\left(p_{deployed}\left(s_{i}\right),p_{original}\left(s_{i}\right)\right)}{\gamma \left(p_{original}\left(s_{i}\right)\right)-\gamma \left(p_{deployed}\left(s_{i}\right)\right)} & \mathrm{denominator}\;\mathrm{values}\;\mathrm{greater}\;\mathrm{than}\;0\\ \inf & \mathrm{else} \end{array}\right.$$

where the numerator is the moving distance and the denominator is the network coverage gain when the node moves from the original position to the current deployed position.

The constraint conditions of this fitness function are as follows:

The node *s_i_* connects with at least one node in *S_CD_* after it moves to the deployment position:

(20) $$\sum_{s_{j}\in S_{CD}}bool\left(s_{i},s_{j}\right)\geq 1$$

where

(21) $$bool\left(s_{i},s_{j}\right)=\left\{ \begin{array}{l} 1\;d\left(p_{deployed}\left(s_{i}\right),p_{deployed}\left(s_{j}\right)\right)\leq R_{c}^{\max}\\ 0\;d\left(p_{deployed}\left(s_{i}\right),p_{deployed}\left(s_{j}\right)\right)>R_{c}^{\max} \end{array}\right.$$

The deployment position of *s_i_* is in the target area:

(22) $$\begin{array}{l} c_{border}\leq x_{deployed}\left(s_{i}\right)\leq x\left(\mathrm{sink}\right)-c_{border}\\ c_{border}\leq y_{deployed}\left(s_{i}\right)\leq y\left(\mathrm{sink}\right)-c_{border}\\ c_{border}\leq z_{deployed}\left(s_{i}\right)\leq z\left(\mathrm{sink}\right)-c_{border} \end{array}$$

(3) Solving the Fitness Function by PSOA

For the selection of the optimal deployment position of a cluster-in node, the pigeon swarm is initialized on the basis of the fitness function and solution space. A description of pigeon swarm initialization follows.

Rule for initializing the pigeon swarm: Each pigeon represents a possible deployment position of the current calculating node, namely, the coordinates of the position in the target area. Thus, the initial position of each pigeon *j* can be expressed as *X_j_* = [*x*,*y*,*z*]. The solution space of the deployment location may consist of multiple intersecting spheres. Thus, the same amount of pigeons is produced in every sphere because pigeons search for the solution space thoroughly to obtain the optimal solution as much as possible. On the assumption that |*S_CD_*| nodes whose deployment positions have been calculated in the cluster of *s_k_*, the rule for producing the initial population is as follows: *N_p_*/|*S_CD_*| random points (i.e., the position of the pigeon) are produced in each sphere, with each node in *S_CD_* as its center and 1.25 $R_{c}^{\max}$ as its radius; all the random points form the initial population of the pigeon. The initial speed of the pigeon is derived randomly in the interval 0−*V_max_*.

The steps to derive the optimal solution by PSOA are as follows:

(1) The pigeons are initialized. The *N_p_* initial positions of the pigeons are determined using the rule for initializing the pigeon swarm.
(2) The fitness of each individual is calculated. The fitness values are sorted according to the following rule: the fitness values of the individuals beyond the solution space are less than those in the solution space. The individual with the minimum fitness is selected as the global optimal solution, *X_g_*, in the current iteration. When several individuals have the same minimum fitness, the individual with the minimum distance to the sink node is selected as *X_g_*.
(3) The position of the pigeon is updated using Equations (8) and (9), the fitness value is calculated, and *X_g_* is updated according to Step (2).
(4) If the value of the current iteration is not equal to the maximum number of iterations, *N*_1_, then Step (3) is repeated.
(5) The solution space based on Equations (10)–(12) is searched locally, and *X_g_* is updated.
(6) If the number of iterations is not *N*_2_, then Step (5) is repeated.
(7) *X_g_* is the deployment location of node *s_i_*.

When *s_k_* has calculated the deployment positions of its cluster-in nodes, each node moves to the corresponding position and broadcasts its new position. The other nodes whose deployment positions are not calculated update their *S_ND_*.

The cluster head node *s_k_* inquires its last-hop node (*s_k_*’*s* child node) whether the deployment positions of its cluster-in nodes have been calculated. If not, the last-hop node of *s_k_* begins to calculate the deployment positions of its cluster-in nodes according to Stage (3) “Optimizing the Position of the Cluster-in Nodes” in Section 4.2.2. Otherwise, *s_k_* transmits the information that the deployment positions of the node in its cluster have been calculated to its next-hop node (*s_k_*’*s* father node), and its next-hop node finds the next cluster where the deployment positions of cluster-in nodes need to be calculated, following the procedure described above in the current paragraph. When all of the clusters in the network have completed the process “Optimizing the Position of the Cluster-in Nodes”, the algorithm ends.

The following Algorithm 1 provides the main steps of the process of optimizing the position of the cluster-in nodes.
**Algorithm 1. Optimizing the Position of the Cluster-in Nodes.****Input:** set of cluster-in nodes *S_c_*(*s_k_*), original position of the cluster-in nodes *P_o_*(*s_k_*). **Output:** deployed position of the cluster-in node *P_d_*(*s_k_*). 1: Initialize* P_d_*(*s_k_*) = *zeros*(|*S_c_*(*s_k_*)|, 3), *S_ND_*, *S_CD_* = *s_k_*; 2: while *S_c_*(*s_k_*) ≠ ∅
3:   calculate the CRR of the node in *S_c_*(*s_k_*); 4:   *s_i_* = the node with the minimum CRR; 5:   *p_original_*(*s_i_*) = [*x_original_*, *y_original_*, *z_original_*] according to *P_o_*(*s_k_*); 6:   assuming that *p_deployed_*(*s_i_*) = [*x_deployed_*,*y_deployed_*,*z_deployed_*] and build the fitness function; 7:   initialize *N*_1_, *N*_2_, *N_p_*, *G*, and the search range; 8:   initialize the position *X_j _*and the speed *V_j_* of each pigeon individual *j*; 9:   calculate the *fitness*_2_ of each pigeon individual; 10:   *X_g_* = arg *min*[*fitness*_2_(*X_j_*)]; 11:   for *N_t_* = 1 to *N*_1_ do 12:     for i = 1 to *N_p_* do 13:       calculate *V_i_* and *X_i_* according to Equations (8) and (9); 14:     end for 15:     evaluate *X_i_*, and update *X_g_*; 16:   end for 17:   for *N_t_* = 1 to *N*_2_ do 18:     if *N_p_* > 1 19:       rank the* fitness*_2_; 20:       *N_p_* = *N_p_*/2; 21:        removed the half of pigeons with a lower *fitness*_2_; 22:       calculate *X_c_* according to Equation (11); 23:       for i = 1 to *N_p_* do (remaining pigeons) 24:         calculate *V_i_* and *X_i_* according to Equation (12); 25:       end 26:        evaluate *X_i_*, and update *X_g_*; 27:     end if 28:  end for 29:  record *X_g_* into *P_d_*(*s_k_*), namely, update the value of corresponding row in *P_d_*(*s_k_*); 30:  *S_c_*(*s_k_*) = *S_c_*(*s_k_*) − *s_i_*; 31:  *S_ND_* = *S_ND_*
∪
*s_i_*; 32:  *S_CD_* = *S_CD_*
∪
*s_i_*; 33: end while;
Notice: *P_o_*(*s_k_*) and *P_d_*(*s_k_*) are the matrix with the size |*S_c_*(*s_k_*)| × 3, and *P_o_*(*s_k_*) = [*P_original_*(*s*_1_); *P_original_*(*s*_2_);⋯,
*P_original_*(*s_i_*)]. In addition, *zeros*(|*S_c_*(*s_k_*)|, 3) is the matrix whose element is zero and whose size is size |*S_c_*(*s_k_*)| × 3.

## 5. Algorithm Analysis

This section analyzes the time complexity of the algorithm. The relevant parameters are presented in Table 1.

### 5.1. Time Complexity of PSOA

PSOA comprises the map and compass operator model and the landmark operator model. During the map and compass operator, each pigeon updates its position according to the global optimal solution, *X_g_*, produced in the last iteration, and subsequently *X_g_* is updated in each of the iterations. The execution time of each of the iterations is 2*N_p_*. Thus, the model’s time complexity, *complexity*_1_, is 2(*N*_1_*N_p_*).

In each of the iterations during the landmark operator, the algorithm initially sorts the pigeons according to their fitness values to calculate the center position, *X_c_*. The execution time of this process is *N_p_* log *N_p_*. The algorithm then updates the position of each pigeon according to *X_c_* and updates *X_g_*; the execution time of this process is 2*N_p_*. Thus, the execution time of each of the iterations is *N_p_*log*N_p_* + 2*N_p_*, and the model’s time complexity, *complexity*_2_, is formulated as follows:

(23) $$complexity_{2}=N_{2}\cdot \left(N_{p}\log N_{p}+2N_{p}\right)$$

The time complexity of PSOA (*complexity*_PSOA_) is

(24) $$\begin{array}{ll} complexity_{\mathrm{PSOA}} & =complexity_{1}+complexity_{2}\\ & =N_{2}N_{p}\log N_{p}+2N_{p}\left(N_{1}+N_{2}\right) \end{array}$$

### 5.2. Time Complexity of PSA

At the stage of network layering and clustering, the nodes broadcast layer by layer for network layering, and the number of layers is not better than *size*/$R_{c}^{\min}$. In each layer, the nodes select the cluster head node synchronously. The worst situation in this process is that a node waits for all its neighbor nodes to give up the competition one by one. The time complexity is *N_a_*. Thus, this stage’s time complexity, *complexity*_3_, is *N_a_*·*size*/$R_{c}^{\min}$.

At the stage of optimizing the positions of the cluster-in nodes, for each cluster, the algorithm firstly selects the node one by one to calculate their CRR, and then calculates the deployment location of each cluster-in node by using the PSOA iteratively, that is, the algorithm uses PSOA to calculate the deployment positions of cluster-in nodes one by one. Thus, for each cluster, the time complexity is *N_ci_* (*complexity*_PSOA_ + *N_ci_*). Assuming that network has *N_c_* cluster head nodes, this stage’s time complexity *complexity*_4_ is formulated as follow:

(25) $$\begin{array}{ll} complexity_{4} & =N_{c}\cdot N_{ci}\cdot \left(complexity_{PSOA}+N_{ci}\right)\\ & =N\cdot \left(complexity_{PSOA}+N_{ci}\right) \end{array}$$

Therefore, the time complexity of PSA, *complexity_PSA_*, is formulated as follows:

(26) $$\begin{array}{ll} complexity_{PSA} & =N_{a}\cdot size/R_{c}^{\min}+N\cdot \left(N_{2}N_{p}\log N_{p}+2N_{p}\left(N_{1}+N_{2}\right)\right)\\ & \leq \left(N_{2}N_{p}\log N_{p}+2N_{p}\left(N_{1}+N_{2}\right)+size/R_{c}^{\min}\right)N \end{array}$$

## 6. Simulation and Performance Analysis

### 6.1. Simulation Scenario and Parameter Settings

This study simulates the node self-deployment process of an underwater wireless sensor network by using the MATLAB simulation platform to analyze the effectiveness of PSA. The target water area (length × width × depth) is set to 120 × 120 × 120 m^3^, the node sensing radius, *R_s_*, is set to 10 m, and the result of each index is calculated as the average value of 30 sets of data. The other parameters are set as follows:

(1) With the method of obtaining the optimal hot spot radius in [34] as basis, the minimum communication radius in this study is set to 12.5 m. The maximum communication radius is set to thrice that of *R_s_* to be consistent with the simulation conditions of TVFDA.
(2) The parameter *G* is set to 0.2, *V_max_* is set to 0.15 times that of the length of the target area, *N_1_*, *N_2_* is respectively set to 35 and 30, and *N_p_* is set to 55 after several experiments on solving the fitness function. (The process is not described in detail in this paper because the experiment is not the point of the problem studied in the paper).
(3) According to [35], the distance between the node and the boundary is 0.866*R_s_* when the full coverage of the network is achieved. This distance is adopted in this study; that is, *c_boarder_* is set to 0.866*R_s_*.
(4) The parameter of the communication energy consumption model and other parameters are set as shown in Table 2.

### 6.2. Simulation

**Figure 5:**
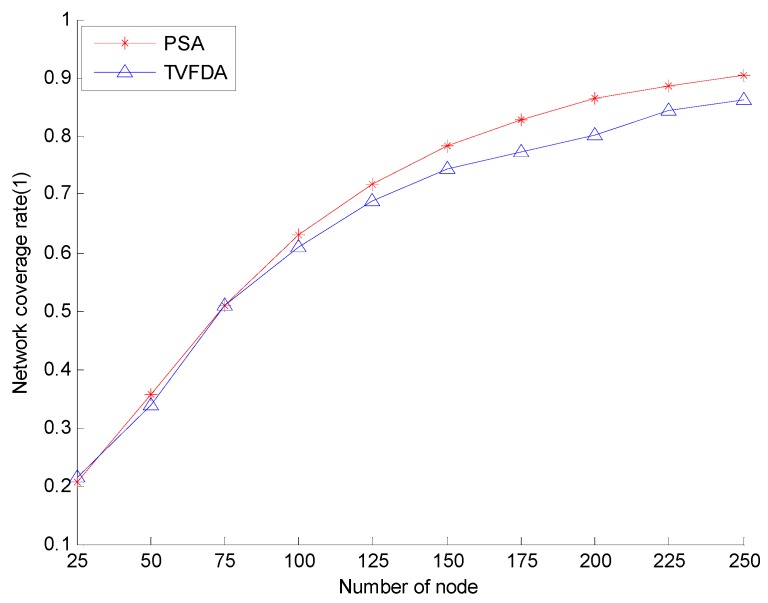
Network coverage comparison.

Figure 5 shows the network coverage rates of PSA and TVFDA with varying number of nodes. The coverage rates of PSA and TVFDA increase with increasing number of nodes. The coverage rates of PSA and TVFDA are highly similar when the number of nodes is lower than 75, but the coverage rate of PSA is higher than that of TVFDA. When the number of nodes is increased, PSA finds more optimal node deployment locations than TVFDA does. This result may be attributed to the process of PSA of determining the moving directions and distances of the nodes by building the optimization model and solving it using PSOA. When the number of the nodes is extremely low, the difference in the results between the two algorithms is not evident. Therefore, when the goal is to improve network reliability and network connectivity even at the risk of reducing network coverage, PSA can still provide network coverage that is slightly higher than TVFDA.

**Figure 6:**
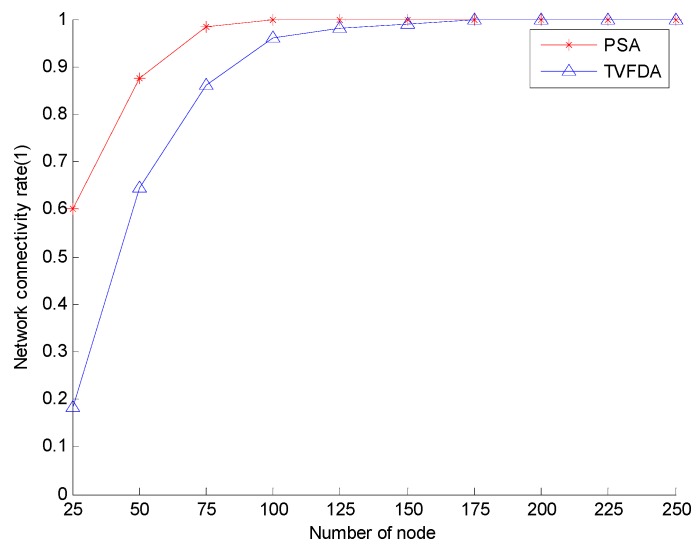
Network connectivity comparison.

Figure 6 shows the network connectivity coefficients of PSA and TVFDA with varying number of nodes. The network connectivity coefficients of PSA and TVFDA increase with increasing number of nodes, and PSA can even reach the state of full network connectivity. Moreover, the network connectivity of PSA is higher than TVFDA when the network nodes are sparse. The superiority of PSA can be attributed to the process of optimizing the node positions by first constructing the backbone of the network and subsequently optimizing the positions of the cluster-in nodes on the condition of maintaining network connectivity. When the network nodes are sparse, all the nodes cannot constitute the backbone network, and the unconnected nodes randomly move a certain distance in the direction of the sink node, thereby increasing network connectivity to a certain extent. By contrast, TVFDA determines the moving direction and distance of each node according to the neighbor of the node, which is very easy to cause the original connected network to be divided and consequently reducing the connectivity of the entire network.

**Figure 7:**
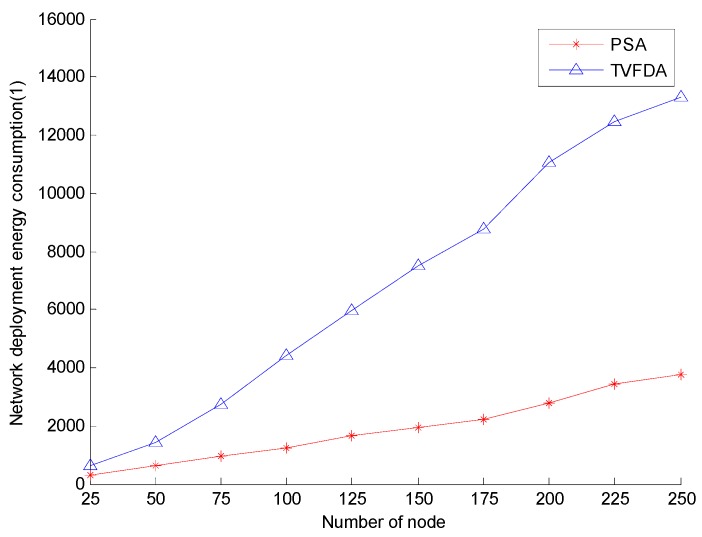
Network deployment energy consumption comparison.

Figure 7 shows the network deployment energy consumption of PSA and TVFDA with varying number of nodes. As shown, the network deployment energy consumption of both algorithms increase with increasing number of nodes, but the network deployment energy consumption growth of TVFDA is significantly greater than that of PSA. In TVFDA, almost all the nodes in the algorithm have to move corresponding distances several times according to the virtual forces exerted on them; thus, the total distance of the node during the network deployment process significantly increases. In PSA, the nodes only move once during the execution of the algorithm, and the network deployment energy consumption is considered when calculating the deployment locations of the cluster-in nodes. In addition, when the network nodes are sparse, a node has no neighbors with large probability, that is, some nodes may not move when TVFDA runs, thereby reducing the total moving distance to a certain extent. Thus, the deployment energy consumption of TVFDA is approximate to PSA when the number of nodes is low.

**Figure 8:**
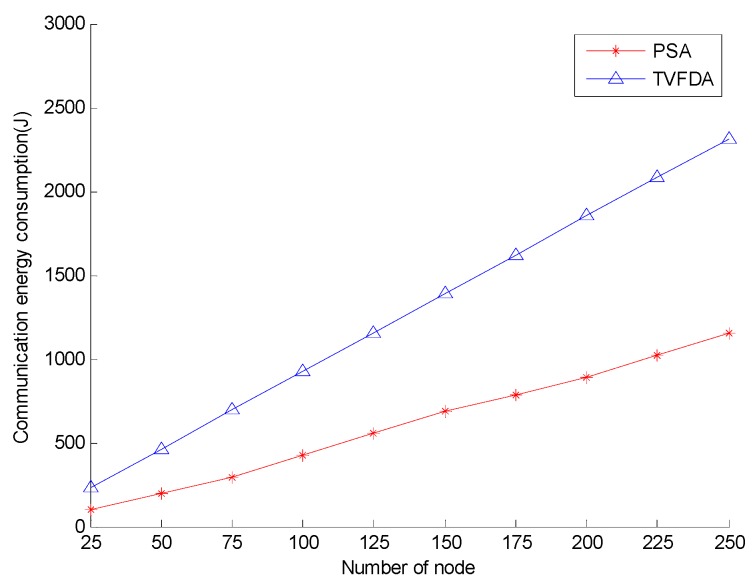
Network communication energy consumption comparison.

Figure 8 shows the communication energy consumption of PSA and TVFDA with varying number of nodes. As shown, the communication energy consumption of PSA is lower than that of TVFDA. TVFDA needs to run several iterations in a distributed manner to improve the network coverage and connectivity, and each node in each of the iterations needs to broadcast a message to obtain the environment information. (According to many experiments, TVFDA needs to run approximately 15 times before stabilizing, when the communication radius of the nodes is 30 m and the nodes are deployed in an area of 120 × 120 × 120 m^3^). However, at the network layering and clustering stage of PSA, each node needs to broadcast a maximum of four messages, and the communication radii of all the nodes are less than the maximum communication radius. Moreover, the locations of the nodes in the cluster can be obtained by optimizing the node position, that is, each of cluster-in nodes should broadcast once. Therefore, PSA is better than TVFDA in terms of communication energy consumption.

**Figure 9:**
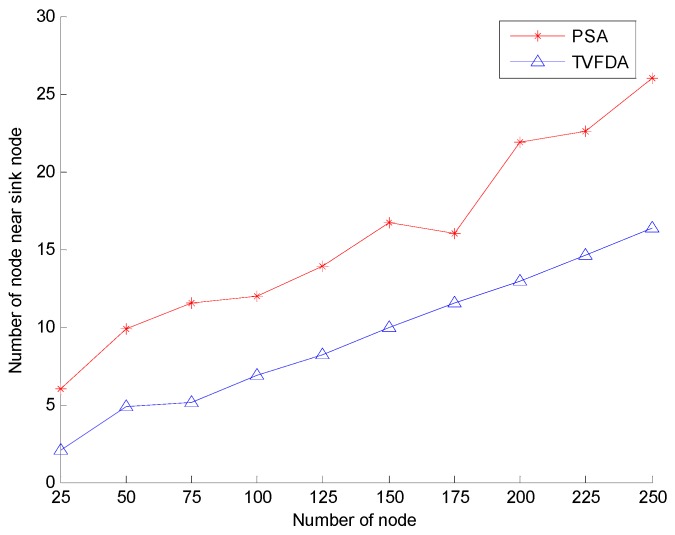
Network reliability comparison.

Figure 9 shows the number of nodes in the region within the distance $R_{c}^{\max}$ to the sink node with varying number of nodes in the entire network. As shown, the numbers of nodes in both algorithms increase. However, for the same number of nodes, the number of nodes in the sink node area after PSA deployment is greater than that after TVFDA deployment. PSA does not optimize the cluster head nodes in the entire network and the number of the cluster head node is greater in the region near the sink node, which increases the number of nodes in the region. However, the objective of TVFDA is to promote the uniform distribution of nodes in the network to increase the coverage of the network without considering the number of nodes in the vicinity of the sink node.

## 7. Conclusions

In this study, we propose PSA for UWSNs to solve the problem in which existing free-to-move node self-deployment algorithms usually target event coverage and cannot improve network coverage under the premise of considering network connectivity, network reliability and network deployment energy consumption. In this algorithm, the sink node first finds its one-hop nodes and maximizes the network coverage in its one-hop region. The one-hop nodes then divide the network into layers and cluster in each layer. Meanwhile, each cluster head node constructs a connected path to the sink node to guarantee network connectivity. The cluster head node finally regards the ratio of the movement distance of the node to the change in CRR as the target function. It employs pigeon swarm optimization to solve the node-deployment problem. The simulation results show that PSA improves both network connectivity and network reliability, decreases network deployment energy consumption, and increases the network coverage.

As a future work, we plan to extend the ideas in this paper considering the node deployment about node probability perception model, which is more in line with the actual situation. The cluster-in position adjustment strategy may need to be modified to some degree. In addition, we plan to consider the additional scenario where the sensor nodes are not anchored. In the scenario, the node may be drifted by the current, and the algorithm should run several times to maximize the network service quality. Thus, the additional trigger mechanism that makes the algorithm run again will be designed.

## Figures and Tables

**Table 1:** Symbol Table.

Parameters	Symbol
Target area size	*size*
Population number	*N_p_*
Iteration number of the map and compass operator model	*N*_1_
Iteration number of the landmark model	*N*_2_
Average number of neighbor	*N_a_*
Average number of non-cluster head node	*N_ci_*
Minimum communication radius	$R_{c}^{\min}$

**Table 2:** Parameter setting.

Parameter	Value
Length of data packet *L_b_*	150 bit
Carrier frequency *F_r_*	24 kHZ
Energy consumption of data reception *P_r_*	20 mW
Data transmission speed underwater *V_t_*	1000 bit/s
